# Supplementary material for: The deubiquitinase USP6 affects memory and synaptic plasticity through modulating NMDA receptor stability
Source: PLoS Biol. 2019 Dec 16;17(12):e3000525. doi: 10.1371/journal.pbio.3000525 (PMC6913916; doi:10.1371/journal.pbio.3000525)

Full gel image of western bloting

Related to Fig 1D

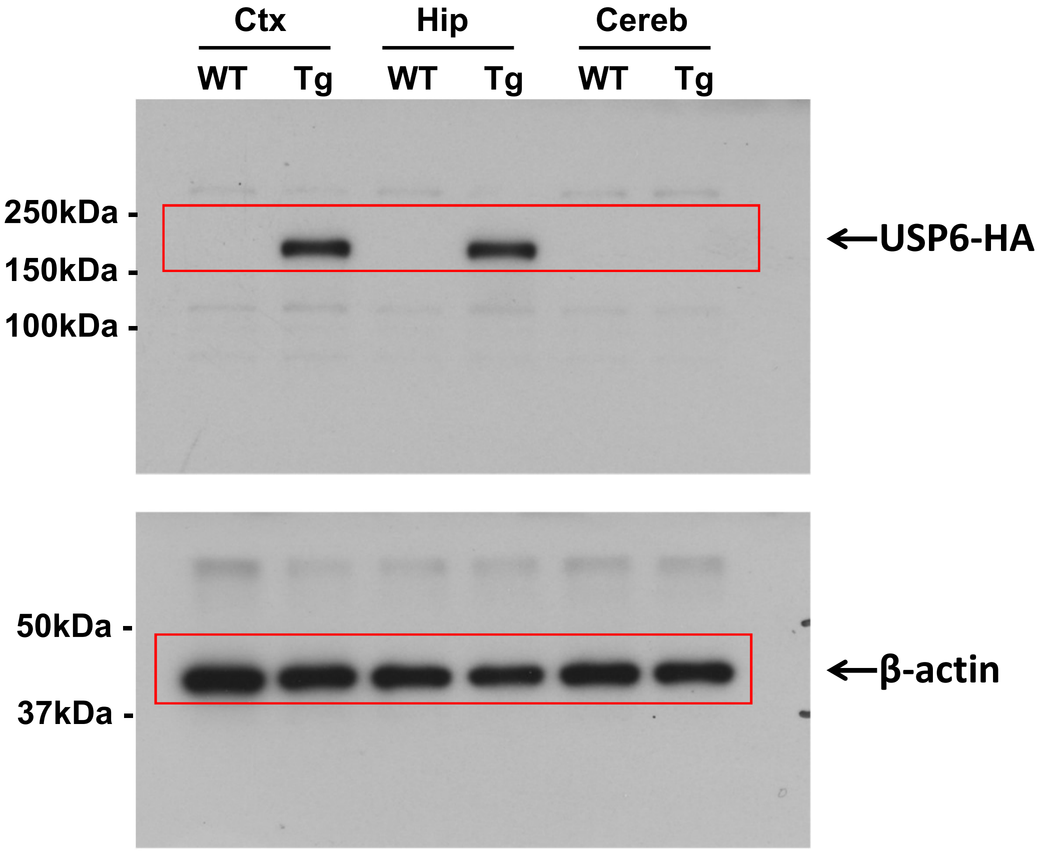

Related to Fig 5A

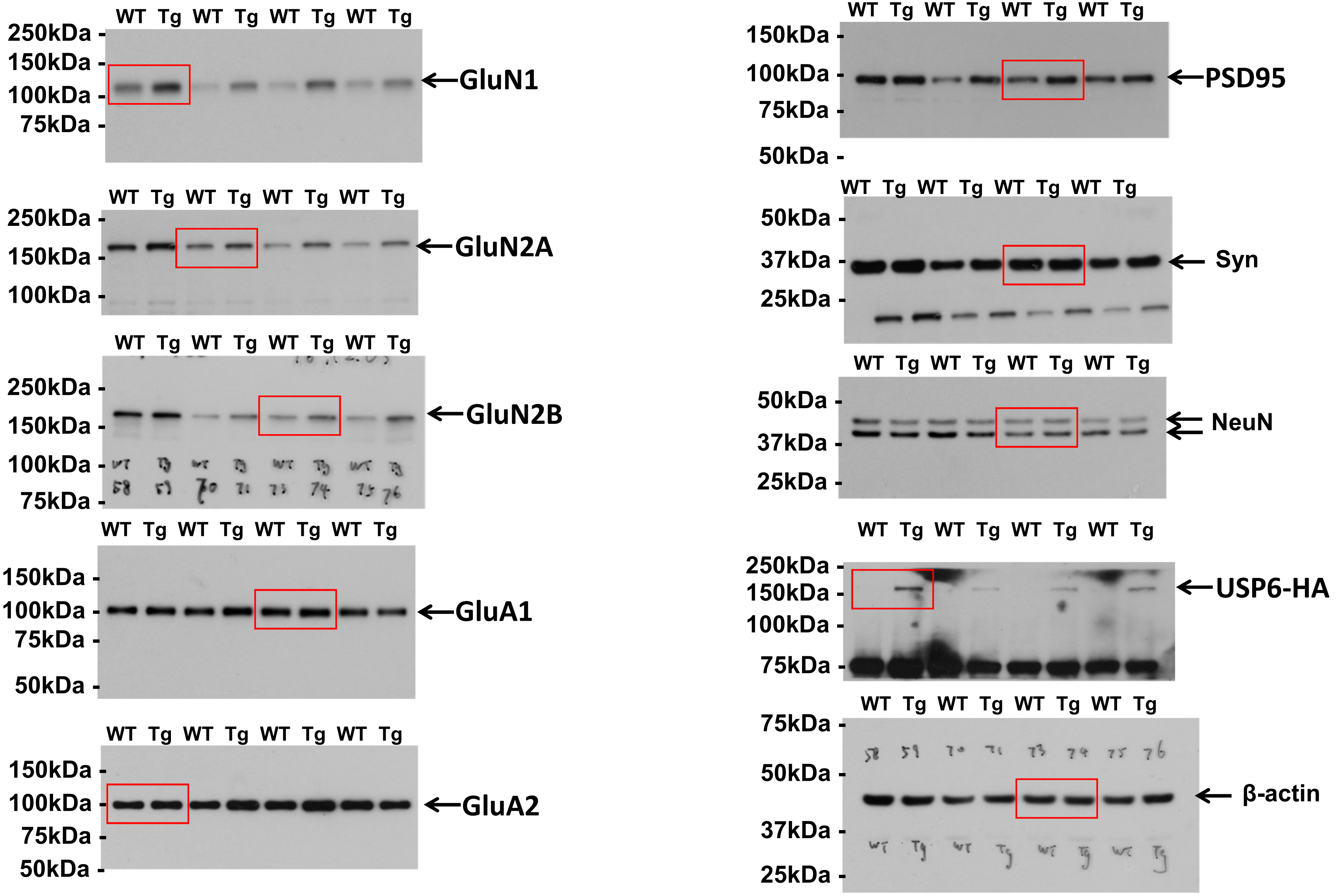

Related to
Fig 5B

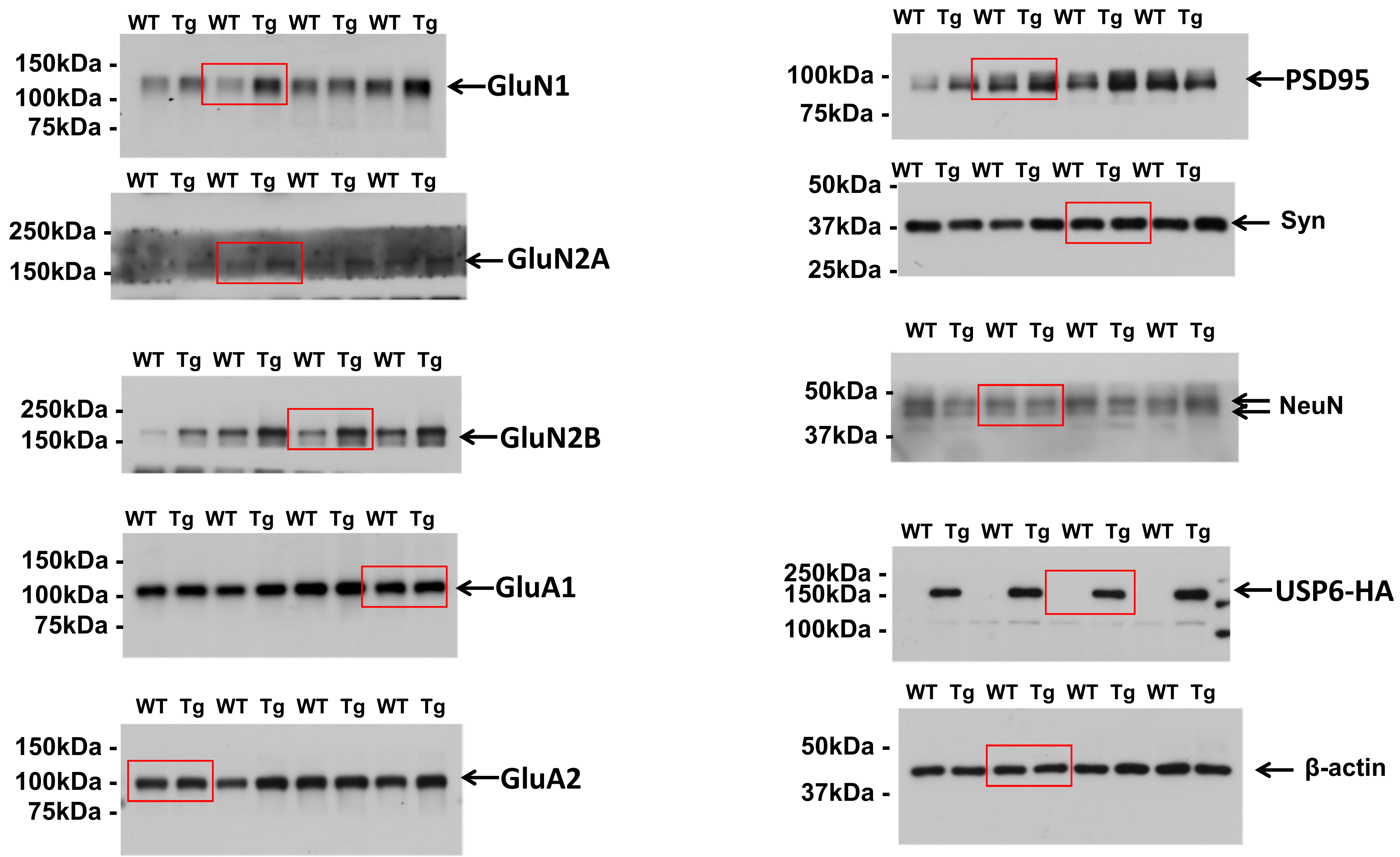

Related to Fig 5C

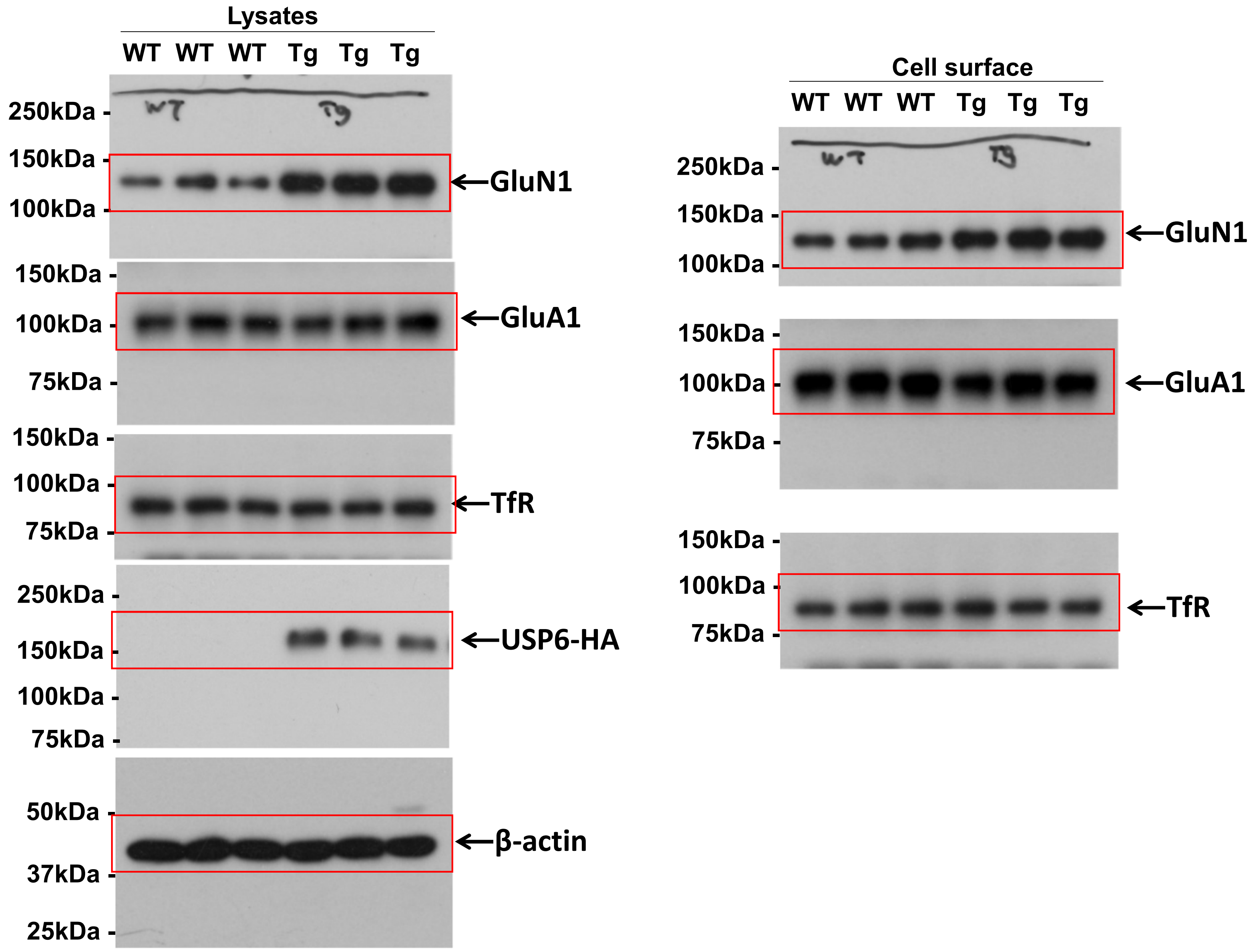

Related to Fig 5D

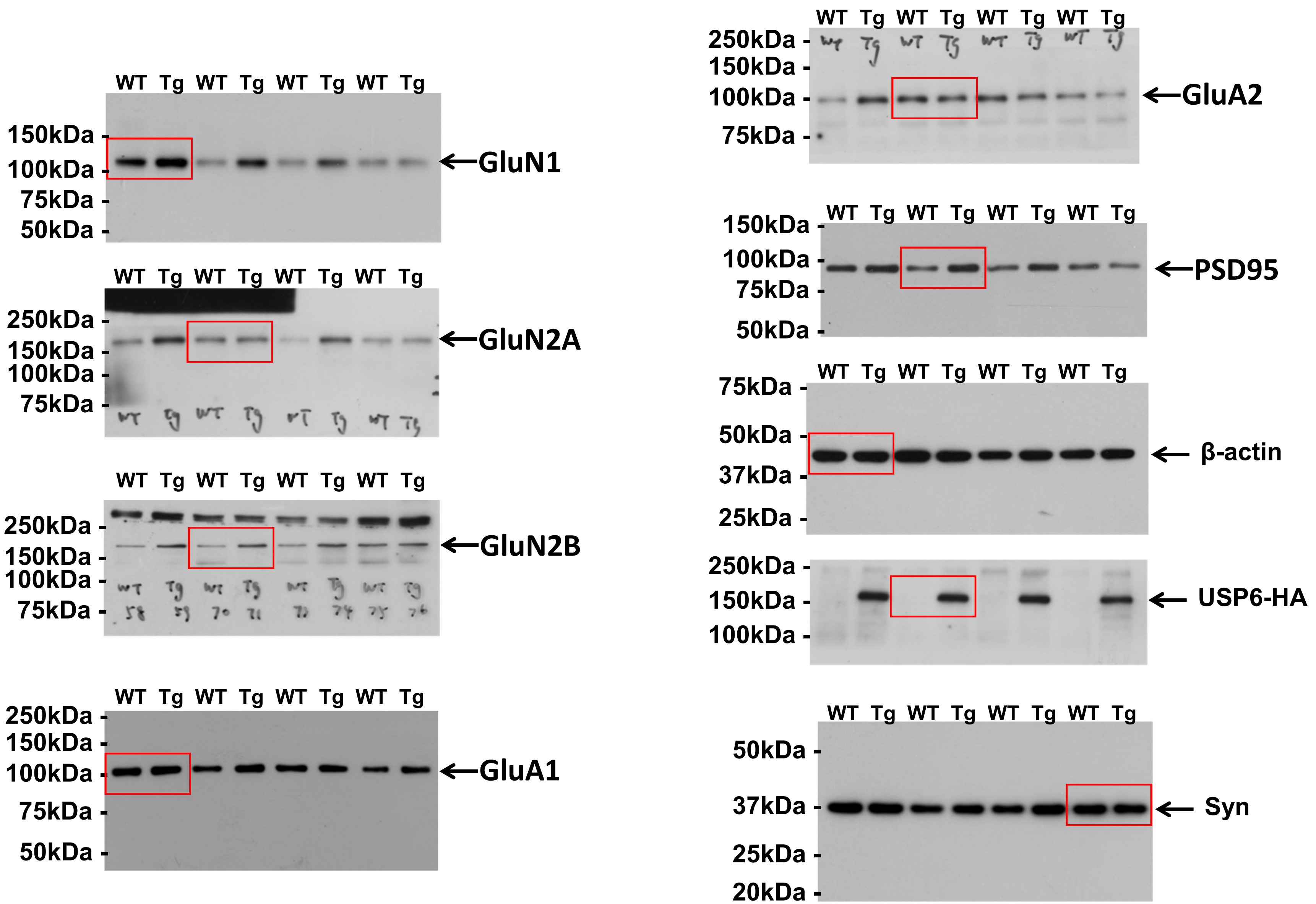

Related to Fig 5D

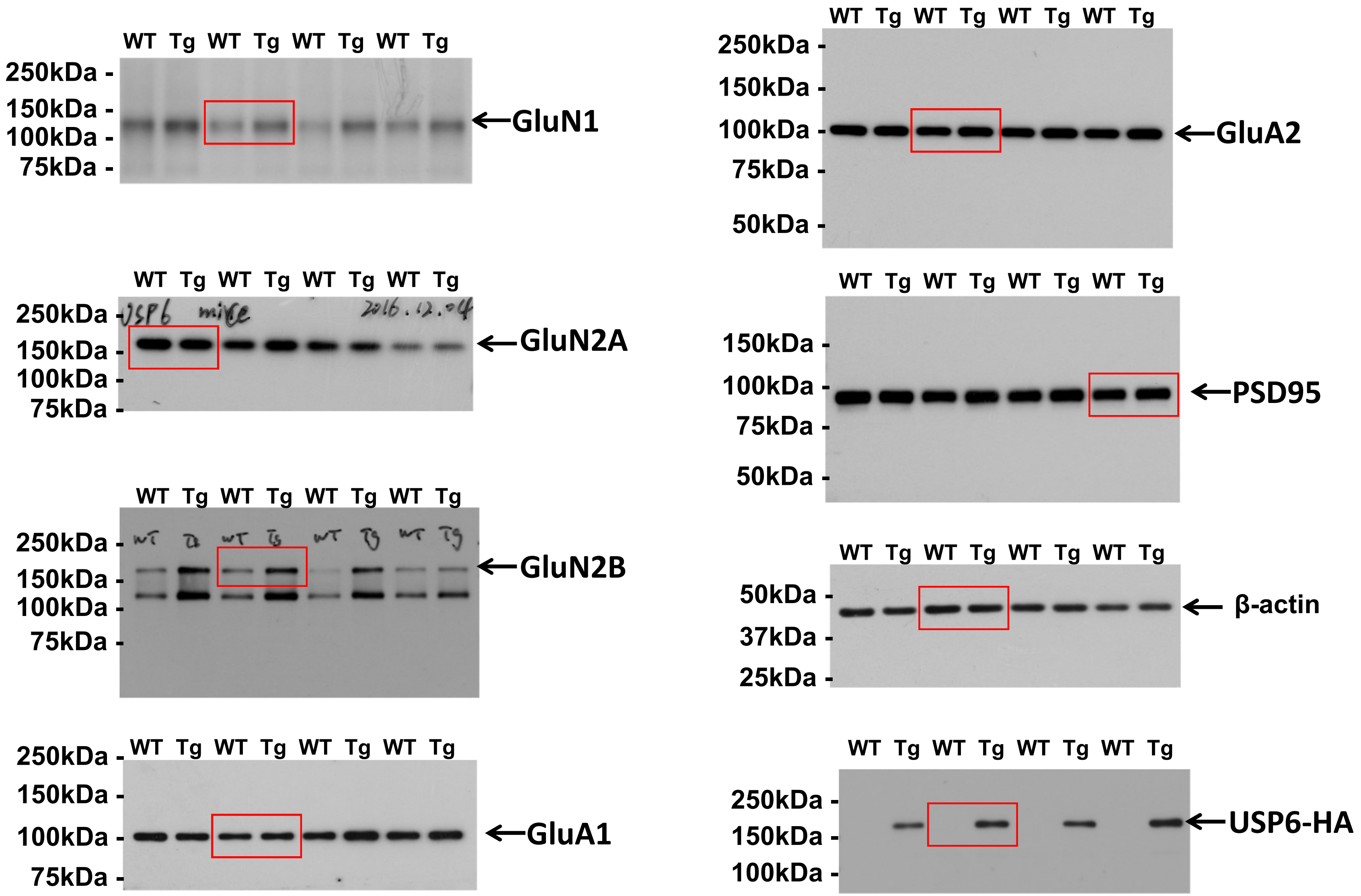

Related to Fig 5E

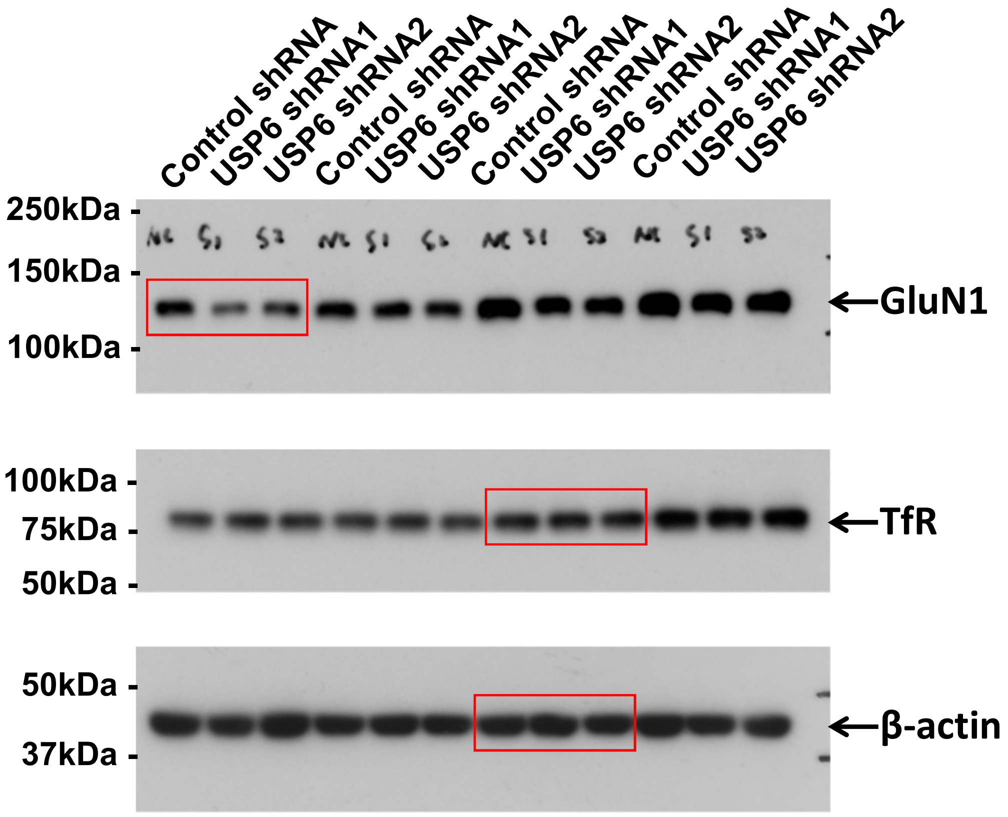

Related to Fig 6A

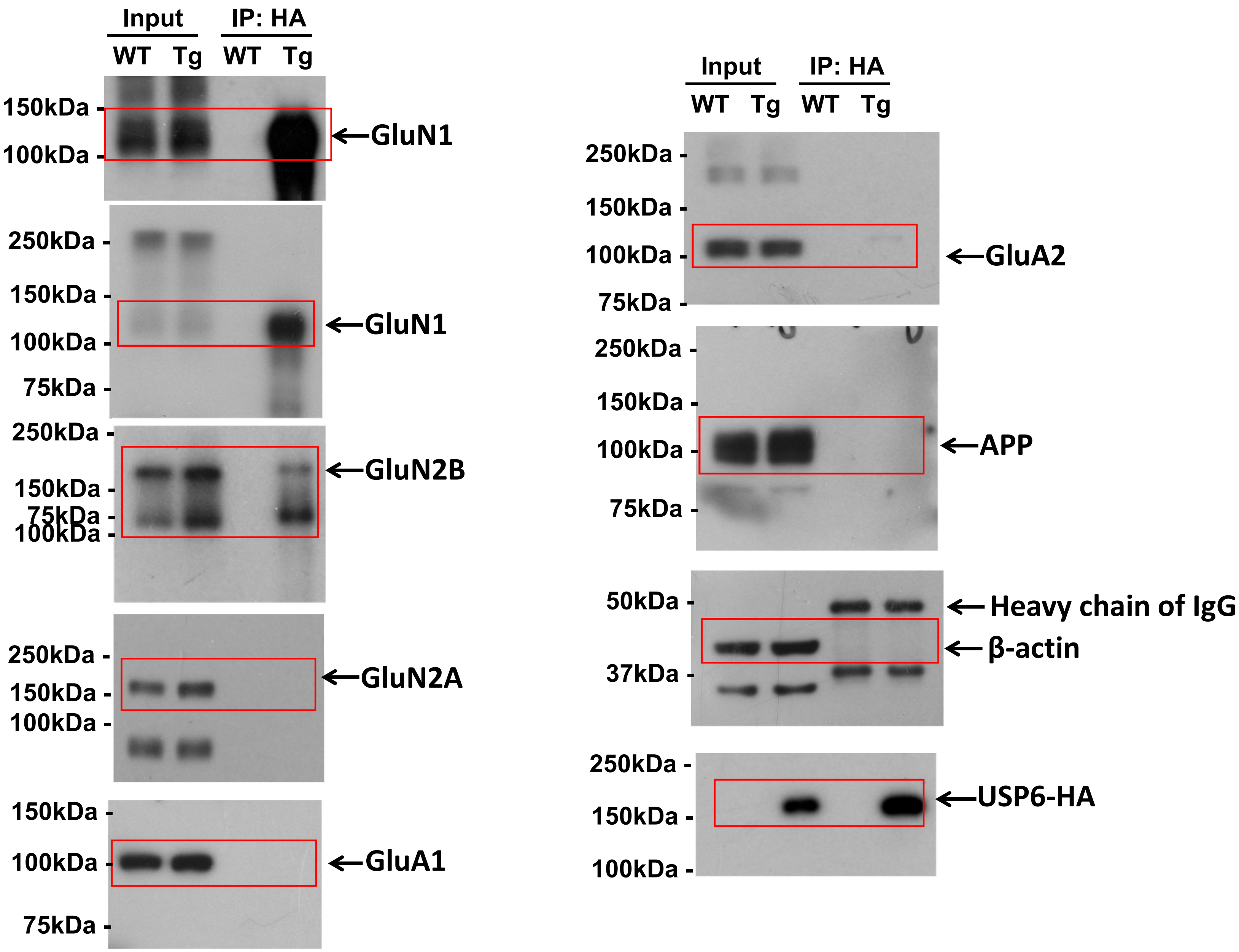

Related to Fig 6B and C

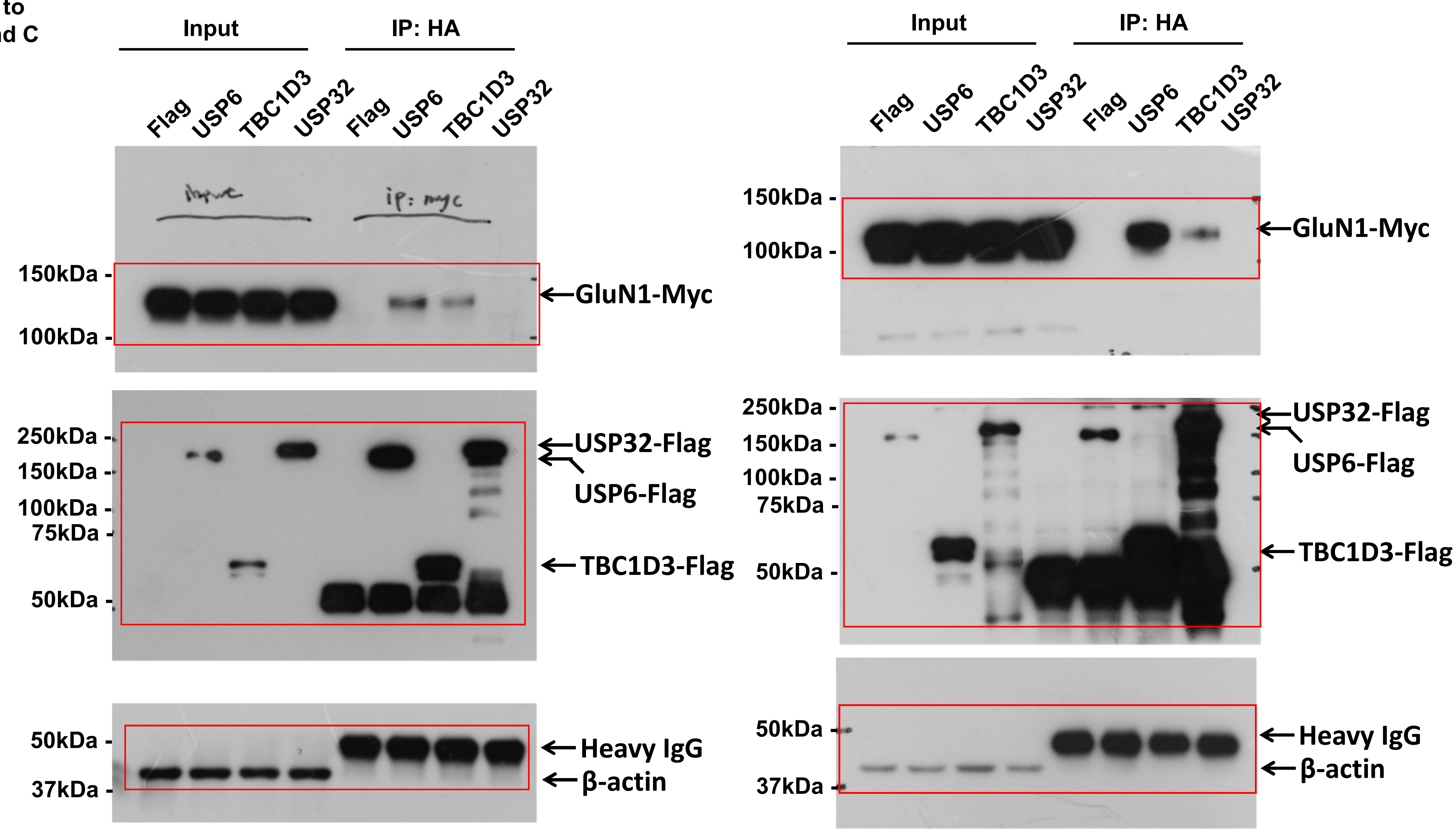

Related to Fig 7A

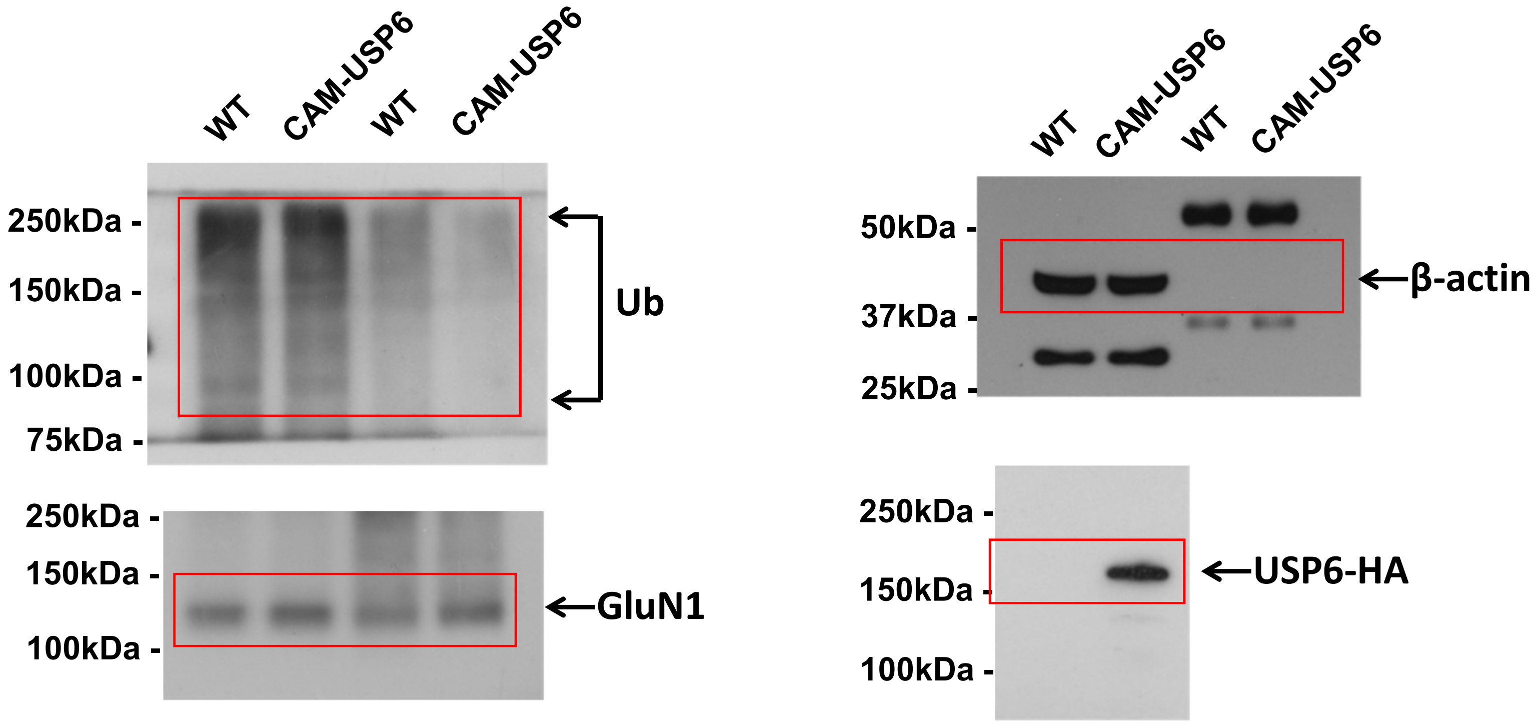

Related to Fig 7B

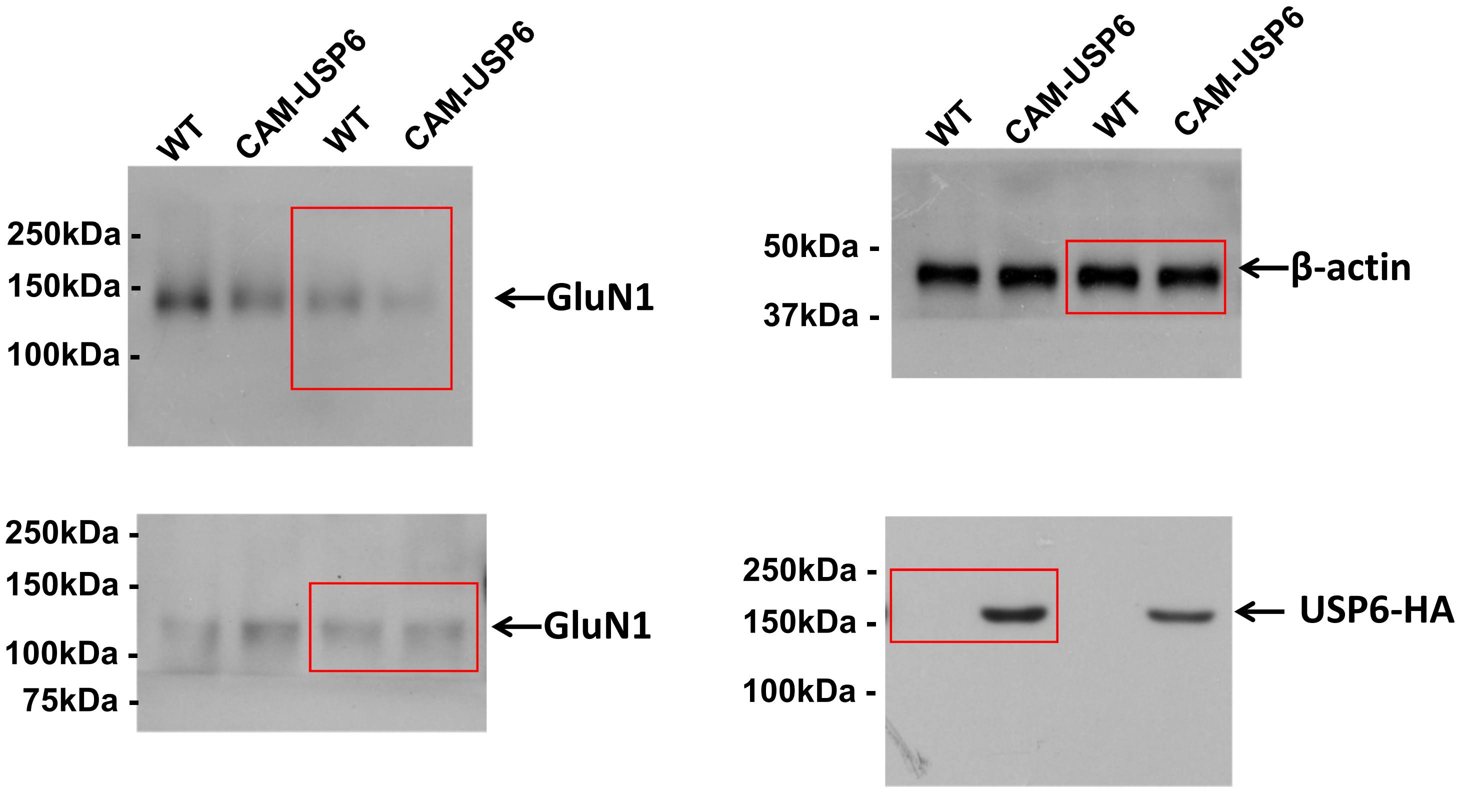

Related to
Fig 7C

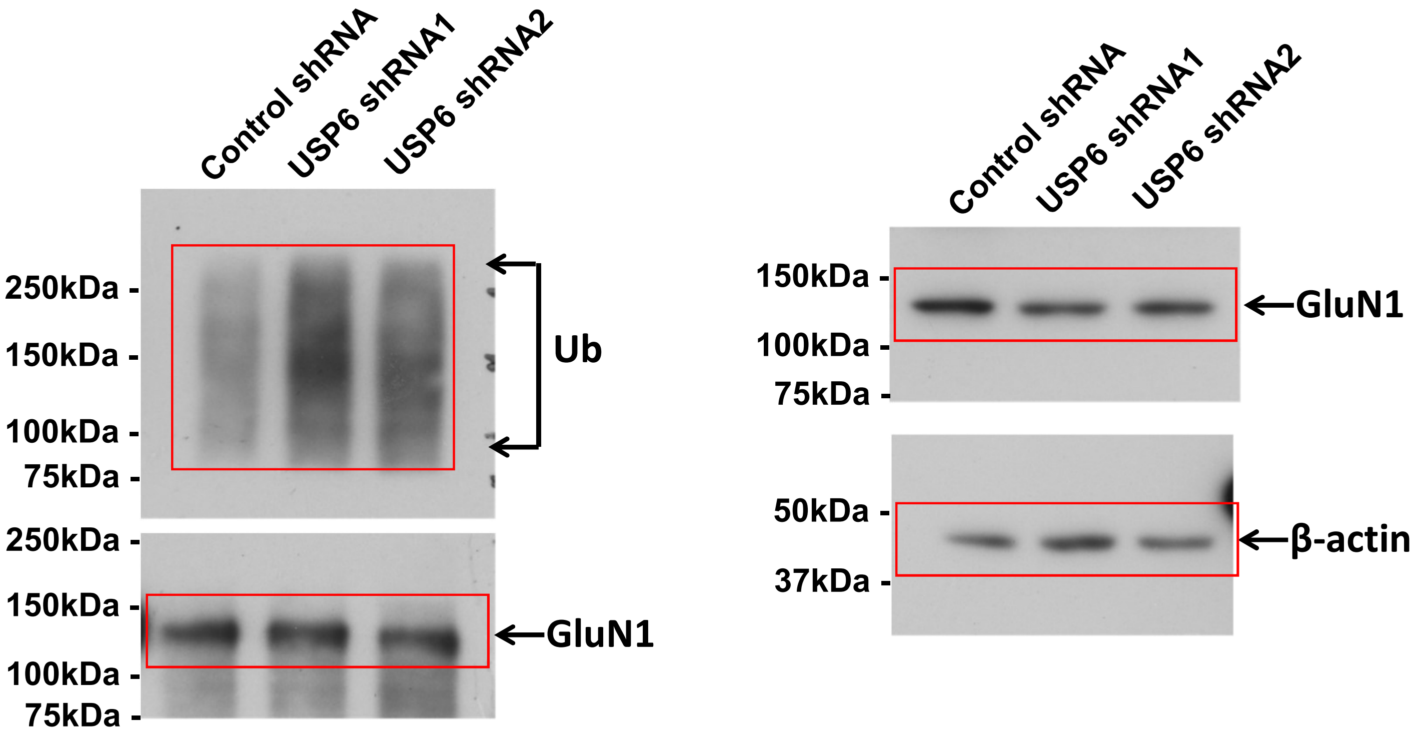

Related to
Fig 7D

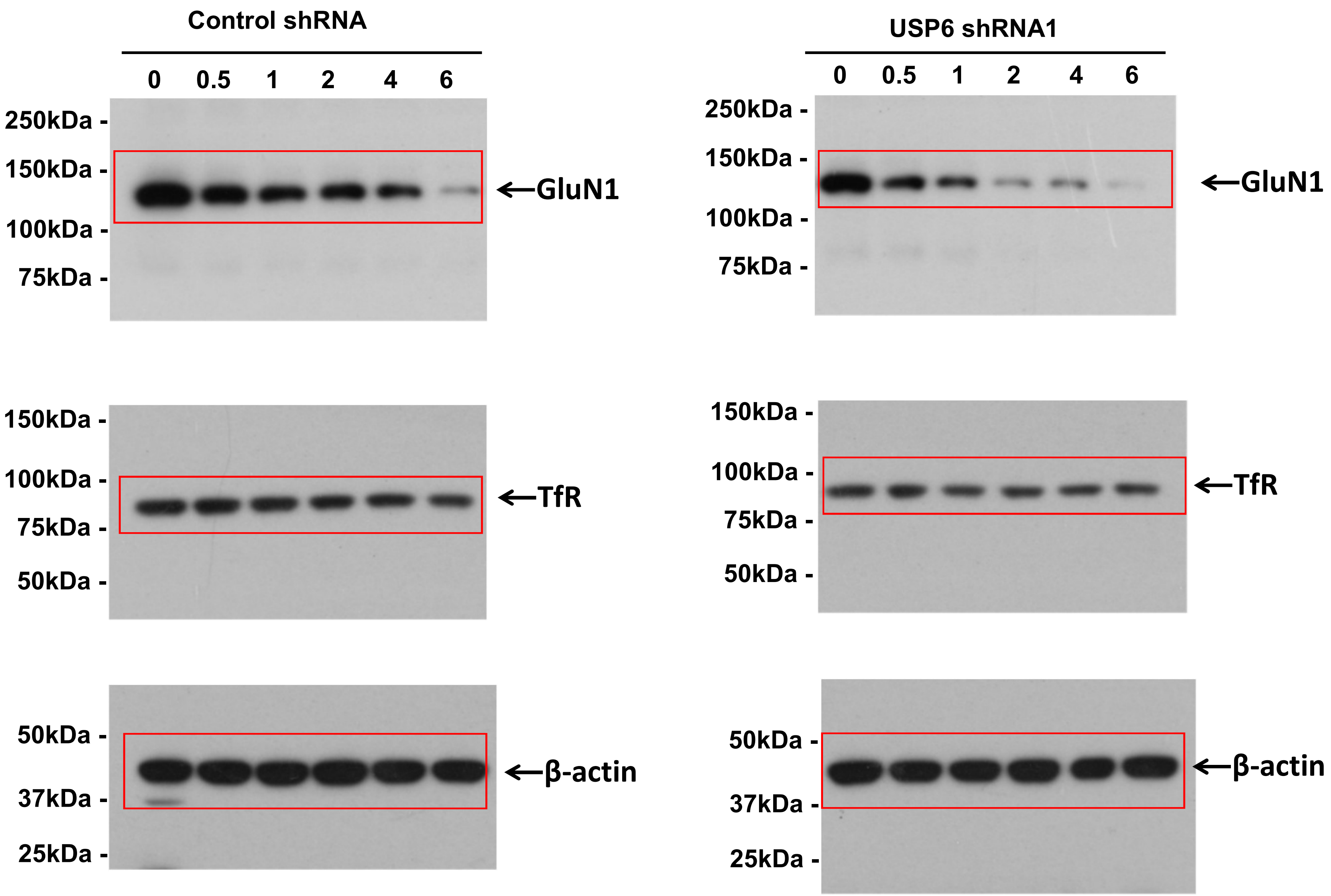

Related to S3B Fig

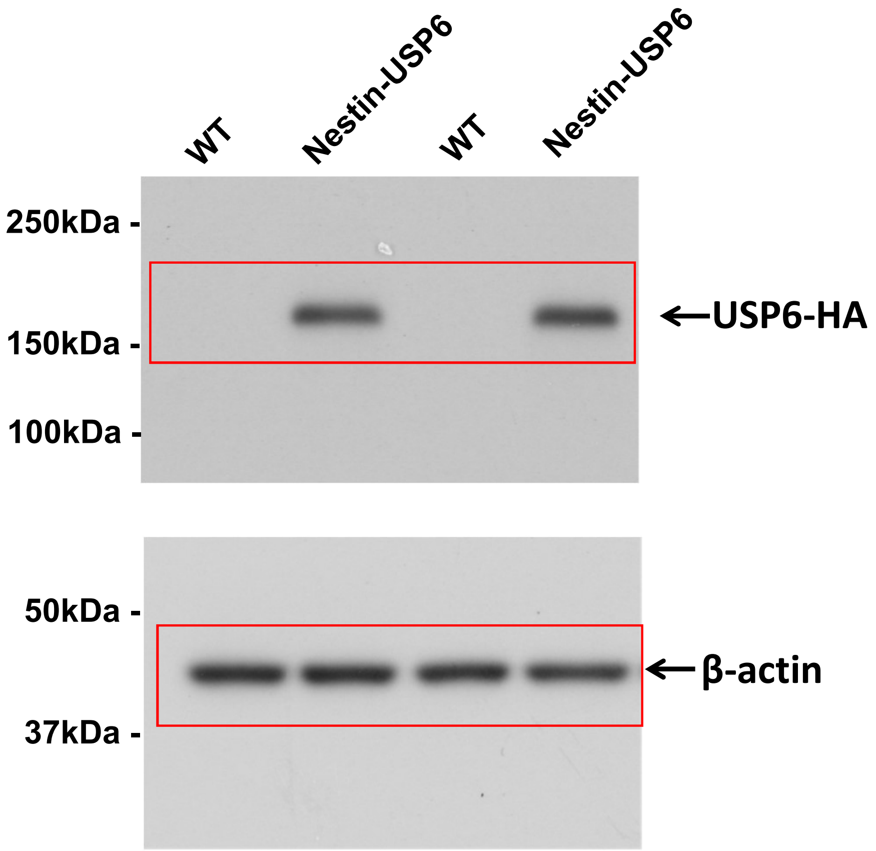

Related to S7 Fig

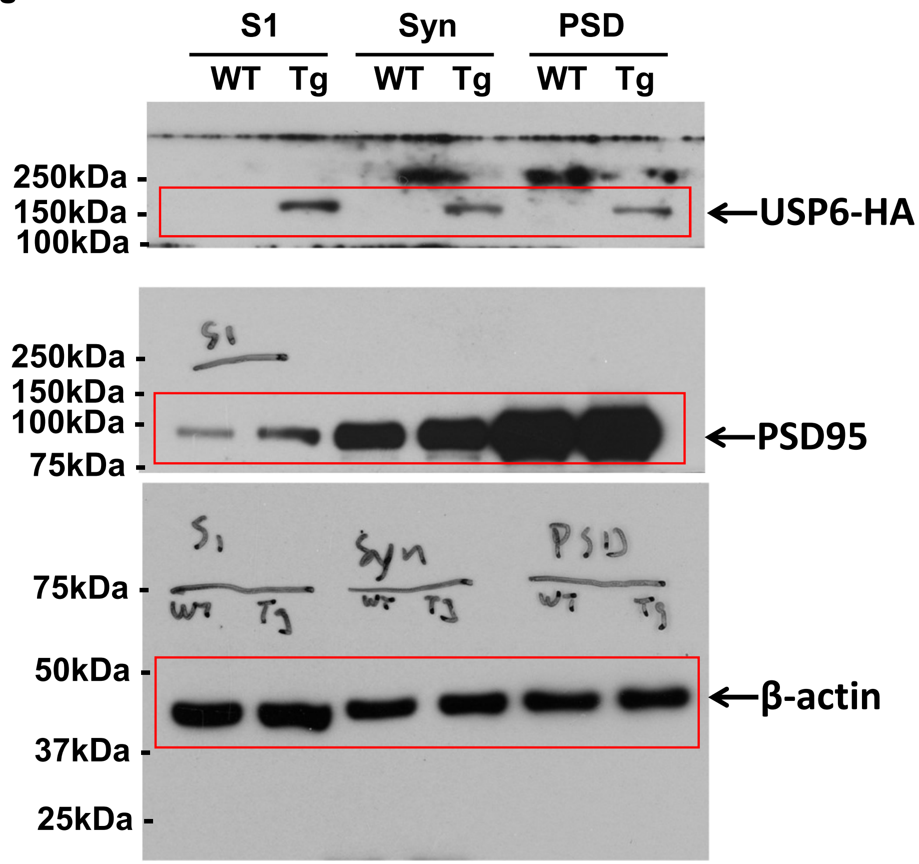

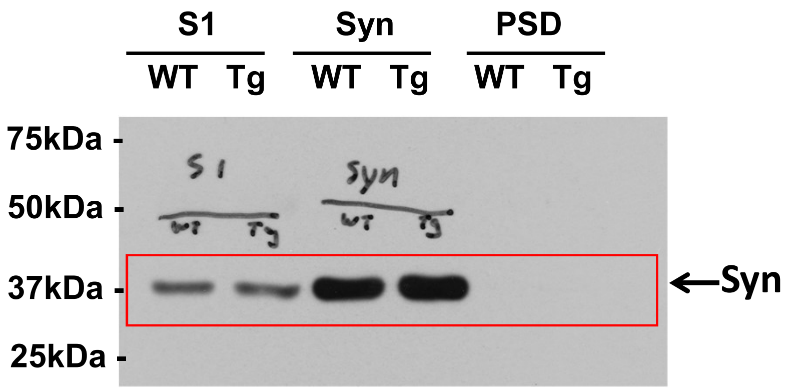

Related to S9A Fig

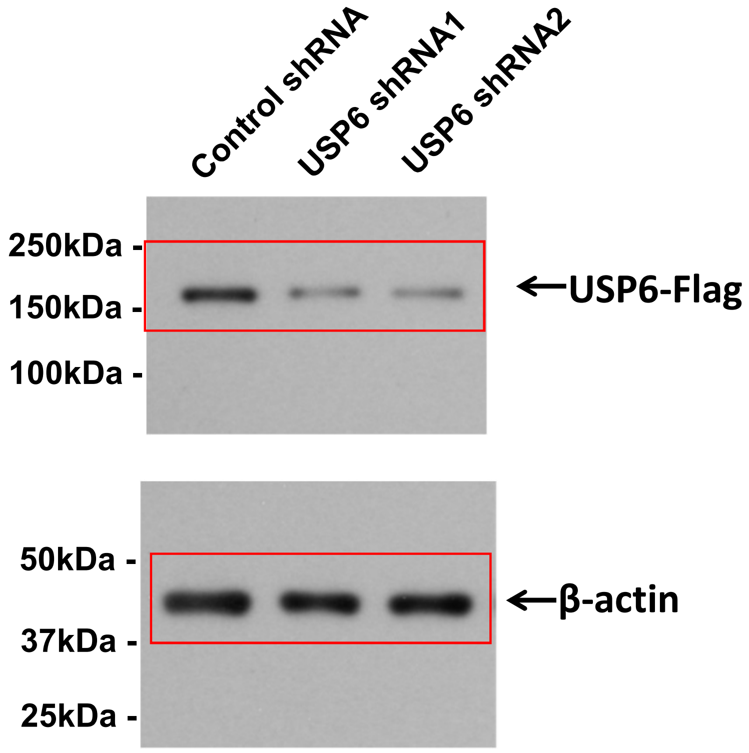

Related to S10C Fig

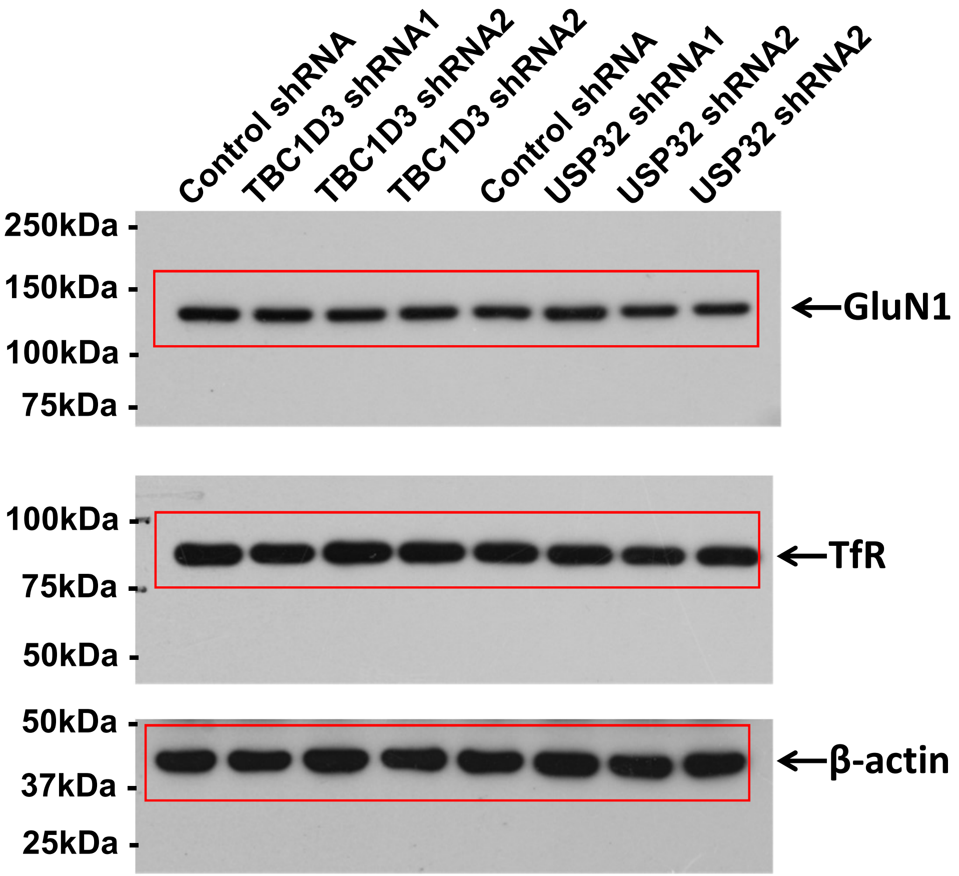

Related to S11 Fig

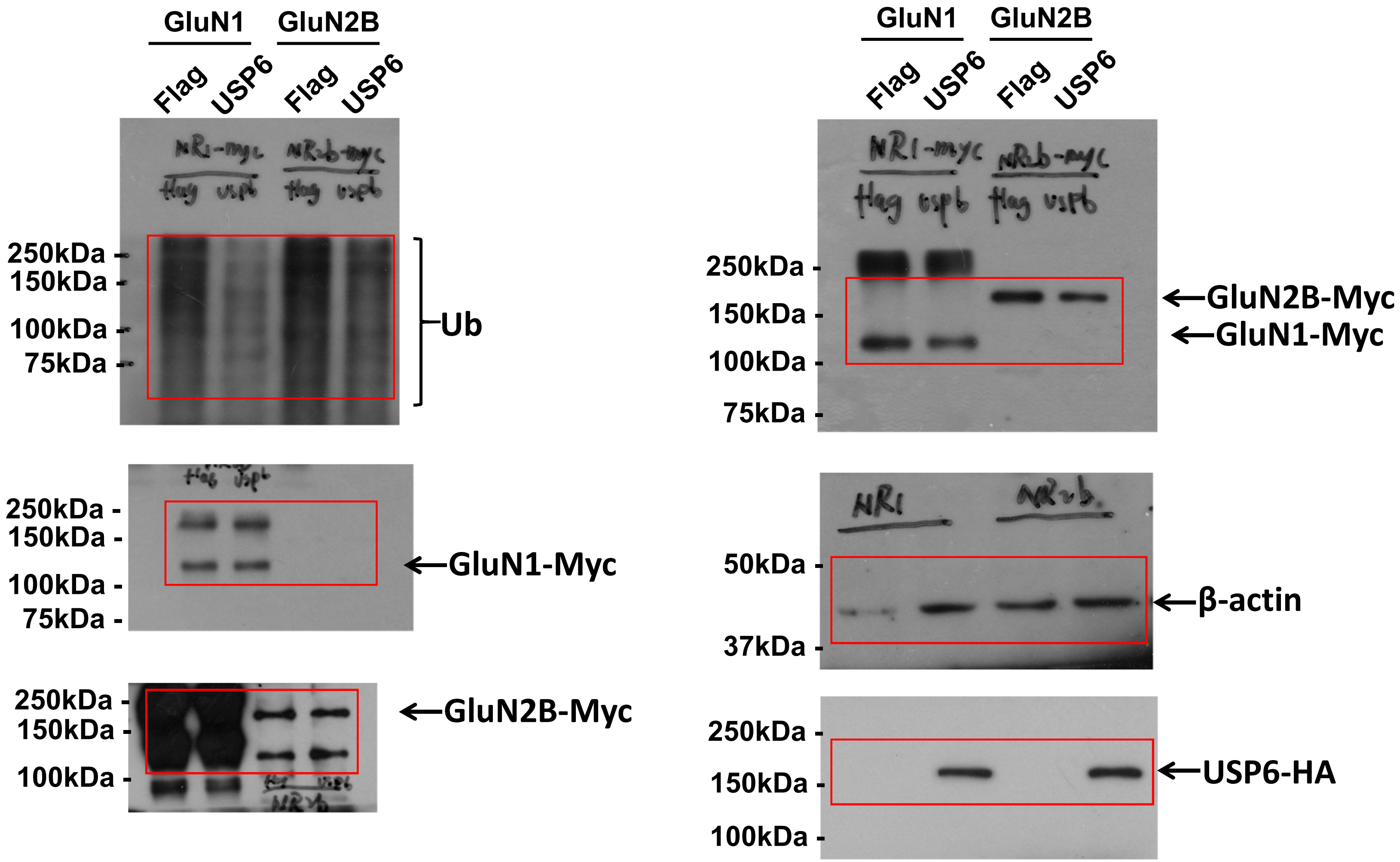

Supplement: S1 Raw Images — The loading order, experimental samples, and molecular weight markers are indicated. The lanes used in the final figure are marked with a red box. (PDF) [file pbio.3000525.s002.pdf]
